# The Intersection of Genitopatellar Syndrome and Oral Health: A Case Report at Saudi Arabia

**DOI:** 10.1155/crid/5053711

**Published:** 2025-08-13

**Authors:** Sara Alzanbaqi, Ahmed Ghibban, Zuhair S. Natto

**Affiliations:** ^1^Department of Pediatric Dentistry, King Saud Hospital, Ministry of Health, Qassim Health Cluster, Unaizah, Saudi Arabia; ^2^Department of Dental Public Health, Faculty of Dentistry, King Abdulaziz University, Jeddah, Saudi Arabia

**Keywords:** genitopatellar syndrome, molar, oral, oral manifestations, pathology, Saudi Arabia, tooth eruption

## Abstract

Genitopatellar syndrome (GPS) is a rare genetic disorder characterized by a spectrum of clinical manifestations including the absence of patellae, psychomotor retardation, congenital flexion deformity of the lower limbs, and genitourinary abnormalities. A 5-year-old female presented to the Faculty of Dentistry Clinic for a routine dental examination. Physical examination revealed distinctive phenotypic features, notably wide thumbnails and limb wrinkling, while facial appearance appeared within normal limits. At birth, the patient exhibited dysmorphic clubfoot, genital anomalies, bilateral hydronephrosis, and hepatomegaly. Subsequent MRI evaluation disclosed bilateral dysplastic femoral trochlea with lateral patellofemoral dislocation, accompanied by marked tibial rotation and vertical talus. Additionally, bilateral hindfoot valgus deformity and first metatarsophalangeal joint flexion deformity were noted. Molecular analysis using Sanger sequencing identified a de novo heterozygous nonsense mutation (c.4117, p.Glu1373Ter) in the KAT6B gene. Oral examination revealed shortened clinical crowns, absence of caries in the primary teeth, and delayed eruption of the primary canines (Cs) and second molars (Es). Radiographic assessment demonstrated existing primary Es and incisors with delayed eruption. This report elucidates a potential association between GPS and oral manifestations, particularly highlighting delayed eruption of primary Es. Since there is a scarcity of publications addressing the oral and dental manifestations of the syndrome, this clinical case contributes, albeit not specifically, to the diagnosis.

## 1. Introduction

Genitopatellar syndrome (GPS) is an exceedingly rare genetic disorder, estimated to occur in approximately one per one million individuals based on existing literature [1]. The first case was reported by Goldblatt et al. in 1988 [2]. However, with fewer than 20 reported cases worldwide, its incidence remains notably low [3]. The syndrome was first delineated by Cormier-Daire et al., marking a seminal contribution to its recognition and understanding [4]. Although GPS affects both genders, reported cases indicate a predilection toward males [3, 5]. The hallmark features of GPS include absent or underdeveloped kneecaps (patellae) and genital anomalies, often characterized by clitoral hyperplasia or undescended testes [1].

Individuals with GPS typically present with profound intellectual disability and mental retardation compared to their counterparts [3]. Facial dysmorphisms, such as microcephaly, a broad nasal bridge, and a small, retracted chin, are commonly observed clinical manifestations [5]. Hypotonia, resulting in respiratory and feeding challenges, further compounds the clinical picture [1]. Additionally, associated cardiac and renal defects may be present [1].

The underlying etiology of GPS is predominantly genetic, attributed to mutations in the KAT6B gene [6]. KAT6B gene dysfunction disrupts the regulation of genes crucial for growth and development, including those governing skeletal and neurological maturation [1, 6]. Given the systemic nature of the syndrome, encompassing both soft and hard tissue structures such as bone, the potential for extended complications, including delayed tooth eruption or congenital tooth agenesis, warrants consideration [7]. However, comprehensive understanding of the full spectrum of GPS remains limited.

To date, no instances of GPS have been documented in Saudi infants, and the potential oral manifestations of the syndrome remain unexplored. Herein, we present the case of a 5-year-old Saudi girl with GPS, who sought dental evaluation at King Abdulaziz University, College of Dentistry, presenting with delayed tooth eruption as the chief concern. This report contributes to the body of knowledge surrounding GPS and underscores the importance of further investigation into its clinical manifestations, particularly within understudied populations. The elucidation of oral manifestations in GPS may offer valuable insights into its systemic effects and aid in comprehensive patient care and management [8]. Additionally, given the scarcity of reported cases in the Saudi population, this study provides a unique opportunity to expand the understanding of GPS epidemiology and phenotype variability within diverse genetic backgrounds [7]. Further research endeavors are imperative to unravel the intricate pathophysiology of GPS and facilitate tailored therapeutic approaches for affected individuals.

## 2. Case Report

**Figure 1:**
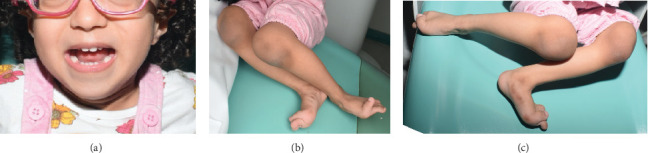
General feature of the patient. (a) An extraoral picture shows no dysmorphic feature, small mouth, pointed, retracted chin. (b, c) Dysmorphic club foot and wrinkled limbs.

**Figure 2:**
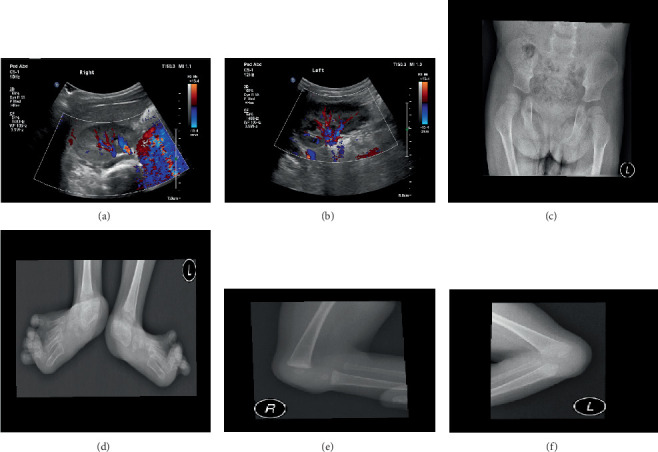
Multiple diagnostic radiographs. (a, b) Ultrasound for the kidney during pregnancy shows hydronephrosis. (c–f) Severe clubbing to the foot and suspected partial metatarsus varus deformity of the left foot with hallux valgus of the first toe, as well as metatarsus varus and clubfoot of the right foot.

A female child, born on August 21, 2019, via full-term (40 weeks) cesarean section with a birth weight of 2900 g (within normal limits), presented to the Pediatric Dentistry Clinic at King Abdulaziz University, Faculty of Dentistry, for evaluation of delayed eruption of her primary teeth, as reported by her parents. She is the third and youngest child of nonconsanguineous Saudi parents; her 10-year-old brother and 7-year-old sister are both healthy, with no family history of congenital conditions. The father's Type II diabetes is well-controlled. Clinical examination revealed wide thumbnails and wrinkled limbs, while her facial features appeared within normal limits (Figures 1a, 1b, and 1c). Intraoral examination indicated the presence of primary teeth without caries, and radiographic assessment confirmed the presence of unerupted primary canines (Cs) and second molars (Es), consistent with delayed eruption. In the medical history, the following features were observed when the baby was born: dysmorphic club foot, abnormality in the genital system, and bilateral hydronephrosis, as well as an enlarged liver. During pregnancy, no abnormality was detected using ultrasound except for the hydronephrosis (Figure 2a,b). An MRI was conducted using multiplanar and multisequential techniques without intravenous gadolinium and revealed no structural brain anomalies and normal appearance of the optic nerve.

An MRI was conducted on both knees and revealed bilateral flattened and dysplastic femoral trochlea with lateral patellofemoral dislocation. The tibia is extremely rotated by almost 90°. The patient had a bilateral vertical talus with dorsal location of the navicular bone, bilateral hind foot valgus deformity, and bilateral flexion deformity of the first metatarsophalangeal joints. The x-ray for her feet displayed severe clubbing to the foot and suspected partial metatarsus varus deformity of the left foot with hallux valgus of the first toe, as well as metatarsus varus and clubfoot of the right foot (Figures 2c, 2d, 2e, and 2f).

The patient was admitted to the hospital at 6 months of age for an MRI under general anesthesia. Later on, the patient was admitted for 3 days because her oxygen saturation had dropped down to 50%. The patient contracted a urinary tract infection with *Escherichia coli* when she was 1 year of age and received a cefixime dose of 30 mg times/day for 11 days. On December 19, 2021, the patient underwent a bilateral operation probing of lacrimal passages under general anesthesia due to a disorder of the lacrimal system and was discharged 1 day after the operation.

**Figure 3:**
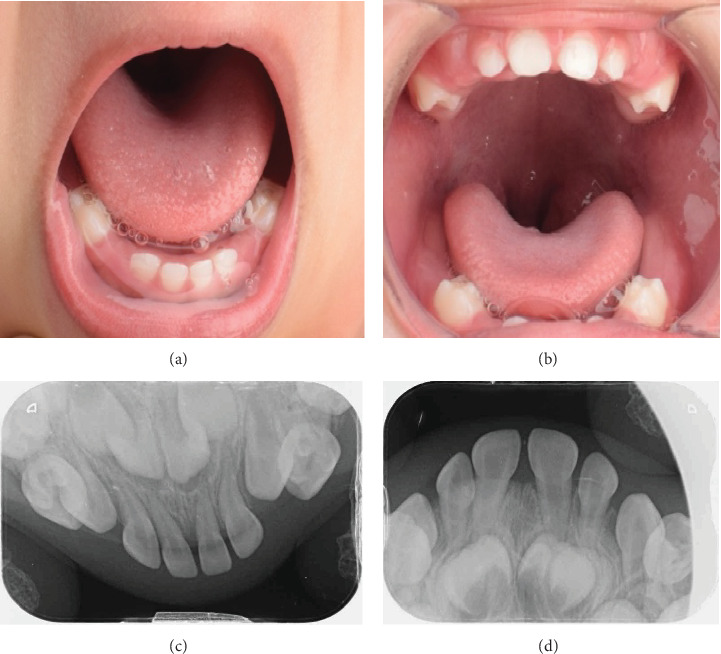
Intraoral pictures and radiographs. (a, b) Intraoral frontal view shows short, small clinical crowns and missing clinically second primary molars and canine. (c, d) Occlusal radiographs show the presence of the second primary molars and canine with delayed eruption.

The oral cavity examination revealed short clinical crowns, caries-free teeth. The incisors and first primary Es are present (54, 52, 51, 61, 62, and 64; 84, 82, 81, 71, 72, and 74). However, the primary Cs and second Es (55, 53, 63, 65, 75, 73, 83, and 85) have not yet erupted, indicating delayed eruption (Figure 3a,b). The upper and lower occlusal radiographs confirmed the presence of all primary teeth, including the Cs and second Es. However, these teeth have not yet erupted, indicating delayed eruption (Figure 3c,d). Based on these findings, the patient does not require any specific dental treatment at this time. We will schedule regular follow-up appointments every 6 months to monitor the eruption progress of her teeth and ensure optimal oral health management.

The ethical research committee at King Abdulaziz University given approval to conduct this case report (#069-05-22). Written, informed consent was acquired from her parents. Genetic analysis was conducted by GenaTi Core Facility Service, which is a Saudi limited liability central laboratory registered with King Abdulaziz University. Using Sanger sequencing–based segregation analysis, we identified a de novo heterozygous nonsense mutation of c.4117 (p.Glu 1373Ter) of KAT6B and normal alleles in the unaffected father and mother.

## 3. Discussion

Several conditions may present with overlapping clinical features and should be considered in the differential diagnosis of GPS. One of the most closely related disorders is Say–Barber–Biesecker–Young–Simpson syndrome (SBBYSS), which is also caused by mutations in the KAT6B gene [9]. While both syndromes share features such as intellectual disability and hypotonia, SBBYSS is often distinguished by more pronounced facial dysmorphism, including blepharophimosis and ptosis, and typically lacks the severe skeletal anomalies seen in GPS. CHARGE syndrome may also be considered due to its multisystem involvement, particularly with genital and renal anomalies; however, it is usually characterized by coloboma, choanal atresia, and mutations in the CHD7 gene. Coffin–Siris syndrome presents with developmental delay and coarse facial features, but is differentiated by the absence or hypoplasia of the fifth fingernails or toenails and involvement of the ARID1B gene. Campomelic dysplasia, caused by SOX9 mutations, involves skeletal and genital anomalies, but is uniquely associated with bowed long bones and more severe respiratory complications. VACTERL association, a nongenetic condition, may also be considered due to overlapping vertebral, renal, and limb anomalies, though it is typically diagnosed clinically in the presence of additional features such as cardiac defects and tracheoesophageal fistula. Despite these similarities, the presence of a pathogenic KAT6B mutation along with the constellation of skeletal, genital, and renal findings supports the diagnosis of GPS in this patient.

Individuals with GPS may experience developmental delays and other systemic issues that could impact oral health due to the underlying genetic abnormalities associated with the syndrome [1, 3, 4]. Although it is difficult to assess the direction of the association, it could be an indirect association due to delays in physical, cognitive, and motor development, which as a consequence can affect the teeth eruption, jaw development, and malocclusion [1, 3]. Moreover, the intellectual disability may affect the ability to maintain good oral hygiene, which can increase the risk of dental caries and periodontal disease [3]. Systemic health issues (heart defects, kidney abnormalities, and skeletal abnormalities) associated with GPS may require medications or treatments that may cause dry mouth or increase the risk of dental caries [1]. The source of the mutation in GPS is predominantly attributed to genetic factors, particularly mutations in the KAT6B gene [6]. These mutations are typically de novo, meaning they arise spontaneously in the affected individual and are not inherited from either parent which is the case in our patient.

To our knowledge, and out of the 20 published cases, there is no case assessing the oral health and teeth eruption of a child with GPS. Lemiere et al. have suggested delayed eruption of primary teeth as a minor criterion for diagnosing the syndrome. However, he did not assess the oral health overall [10]. It could be because the cases died within the first year of age, before the eruption of primary teeth, or at a later stage of life when the permanent teeth eruption time has already passed. The oldest patient with GPS is an almost 30-year-old female without renal or cardiac anomalies [5], followed by a 22-year-old [3]. Few case series/reports have been published where the patient died in early life. Cormier-Daire et al. investigated three patients who died within the first years of life due to sudden death or respiratory collapse. It is worth noting that the oral findings are contrary to what one might expect, as the oral hygiene is good and no caries were found. This is different from other diseases at the same age, such as renal dysfunction, where they have a low caries rate, enamel hypoplasia or hypomineralization, pale oral mucosa, dry mouth, poor oral hygiene, uremic stomatitis, and irregular salivary flow [11–14]. However, there was not a delay in teeth eruption.

The long-term prognosis of these cases is unpredictable, and it depends on the degree of heart, kidney, or respiratory complications. Many cases were suffering from renal impairment, hypergonadotropic hypogonadism, and/or pituitary–adrenal axis dysfunction [4]. In our patient, renal function is still fine, and it is still too early to assess puberty.

The management of patients with complex genetic syndromes such as GPS necessitates a multidisciplinary approach that involves close collaboration between dental professionals and medical health providers. Given the systemic nature of the condition, which often includes renal, orthopedic, neurological, and endocrine involvement, coordinated care ensures comprehensive evaluation and management, minimizing the risk of overlooking critical health issues. Working in alliance with pediatricians, geneticists, and other specialists allows for a more accurate diagnosis, appropriate referral, and safer dental treatment planning, especially when general anesthesia or pharmacologic interventions are considered. Furthermore, obtaining informed consent from medical guardians and ensuring transparent communication with all healthcare providers are ethically essential, as it respects the autonomy of the patient and their family while promoting patient safety. Such interdisciplinary cooperation not only enhances the quality of care but also facilitates early recognition of potential oral manifestations that may otherwise be missed in isolated clinical settings.

## 4. Conclusion

It should be noted that there is a potential slight delay in primary teeth eruption in the molar areas; these results may contribute to understanding the clinical features of GPS cases, particularly regarding the timing and sequence of dental development. Since there is a scarcity of publications addressing the oral and dental manifestations of the syndrome, this clinical case contributes, albeit not specifically, to the diagnosis.

## Data Availability

The data from this study are fully available in this article.
